# Assessing validity of the Klenico diagnostic software system in a large psychotherapeutic inpatient sample

**DOI:** 10.3389/fdgth.2023.1176130

**Published:** 2023-08-24

**Authors:** Stefan Reutimann, Noah Hübscher, Jasmin Steiner, Ulrich Voderholzer, Mareike Augsburger

**Affiliations:** ^1^ Klenico Health AG, University of Zurich Startup, Zurich, Switzerland; ^2^ Department of Health Sciences and Technology, Swiss Federal Institute of Technology, Zurich, Switzerland; ^3^ Schoen Clinic Roseneck, Prien am Chiemsee, Germany; ^4^ Department of Psychiatry and Psychotherapy, Ludwig-Maximilians-University Munich, Munich, Germany

**Keywords:** Klenico, ICD-10, multitrait-multimethod, validation, diagnostic procedures, construct validity, digital mental health

## Abstract

**Introduction:**

Mental disorders are often underdiagnosed in routine diagnostic procedures due to the use of unstandardized assessments; this can result in people either not receiving necessary treatment or receiving ineffective treatment for their condition. Klenico is an online diagnostic software system that facilitates diagnosis of mental disorders in adults through the use of standardized procedures. The procedure encompasses two modules, self-report and clinical validation. The current study aimed to confirm the validity of the Klenico assessment in a large clinical sample.

**Methods:**

Fully anonymized data from 495 adult inpatients were used. ICD-10 diagnoses were made during an initial interview by the clinical staff. Afterwards, patients filled out self-report questionnaires (BDI-II, BSI, EDE-Q, OCI-R, PHQ-D, and Y-BOCS) and completed the Klenico self-report module, which involves selecting and rating the severity of applicable symptoms. Finally, in the clinical validation module, mental health professionals validated the symptoms endorsed in the self-report module. Six Klenico domains were tested against patient self-reports and routine ICD-10 diagnoses by following the multitrait-multimethod approach. Internal consistency was assessed by calculating Cronbach's alpha.

**Results:**

The Klenico depressive disorders, OCD, and somatoform disorders domains revealed high correlations with the congruent questionnaires (i.e., those pertaining to these specific disorders) and revealed low correlations with the noncongruent questionnaires (i.e., those pertaining to other disorders), therefore evidencing construct validity. For the eating disorders and psychotic disorders domains, divergent validity was demonstrated. For the anxiety disorders domain, although analysis mostly indicated construct validity, this should be further confirmed.

**Discussion:**

Overall, the results largely confirmed the construct validity of the Klenico assessment, demonstrating its use as an easy-to-use, valid, standardized, and comprehensive instrument for diagnosing mental disorders.

## 1. Introduction

Mental disorders are widespread across the globe. Estimates suggest that the general population has a 25% 12-month prevalence (1) and that one in three people will develop a mental disorder at some point in their life (2). Mental disorders have profound detrimental effects beyond individual suffering. Patients with mental disorders show significantly higher rates of mortality (3), which results in a substantial reduction in life expectancy of about 10–20 years (4). In addition, mental disorders impose substantial economic costs on the individual as well as on society. While individuals experience less income due to their disability, societies are faced with substantial health care costs and are additionally impacted by the loss of productivity from reduced labor, the loss of income tax revenue, and increased government support payments (5–7).

Further aggravating the burdens on an individual and societal level is the comorbidity commonly seen in mental disorders (8–12). For instance, a representative study with 9,282 adult respondents revealed that in more than 40% of 12-month cases, patients with mental disorders had comorbid diagnoses (12). Comorbidity in mental disorders is of great clinical importance because it is strongly related to overall disorder severity (12). Patients with multiple, comorbid mental disorders are likely to have poorer treatment outcome and prognosis as well as increased levels of suicidality (13, 14).

In routine clinical practice, however, comorbidity is often underrecognized (15) and mental disorders in general are often under- or misdiagnosed (16, 17). For instance, misdiagnosis rate in primary care has been shown to reach up to 66% for major depression (16) and up to 50% for bipolar disorders (17). This underdiagnosis is often the result of the use of unstandardized instruments (18). A meta-analysis based on 39 primary studies with more than 15,000 participants comparing the results of standardized diagnostic interviews and unstandardized interviews by clinical experts demonstrated that a significant portion of diagnoses are missed if unstandardized interviews are used (19). Moreover, diagnostic agreement between the two approaches was only low to moderate for most diagnoses. Consequently, there are considerable differences in the outcome of the diagnostic assessment when interviews are conducted by different methods (19). These findings highlight the importance of standardized diagnostic procedures.

However, in routine clinical practice, standardized instruments are rarely used. For example, only 15% of patients are diagnosed by using structured interviews. One reason for this low use of structured assessments is that mental health professionals consider these too time-consuming. Moreover, familiarity with such instruments is lacking, while clinical utility of unstructured clinical interviews is overestimated (20). For these reasons, a standardized yet time-efficient diagnostic software program, which is easy to implement in everyday clinical practice, could have the potential to fill this gap in current diagnostic procedures.

To address this challenge, Klenico was developed (21). Klenico is an online diagnostic software tool for routine mental health care based on the 10th revision of the International Classification of Diseases (ICD-10) (22). Klenico uses standardized procedures and involves two modules. First, in the self-report module, patients select and rate applicable symptoms. Second, in the clinical validation module, mental health professionals validate the symptoms endorsed in the self-report module by means of a semi-structured interview. Based on the information obtained in these two modules, mental health professionals are guided toward ICD-10 diagnoses.

Klenico covers the following mental disorders and aggregated disorder domains: anxiety disorders [agoraphobia, generalized anxiety disorder (GAD), panic disorder, social pobias, specific phobias]; disorders of adult personality and behavior; eating disorders (anorexia nervosa, bulimia nervosa); mental and behavioral disorders due to psychoactive substance use; mood (affective) disorders (depressive disorders, mania); obsessive-compulsive disorder (OCD); reaction to severe stress and adjustment disorders; and psychotic disorders (schizophrenia, schizotypal and delusional disorders). Additionally, aspects of autism spectrum disorder, attention deficit hyperactivity disorder (ADHD), and dementia were included. Furthermore, Klenico screens for self-harm, potential harm to others, and suicidal ideation.

Klenico was developed in collaboration with clinical experts, including psychologists and psychiatrists, to ensure high content validity. For additional information on how the Klenico system was developed, including the selection of disorders and symptoms, as well as the development and selection of items, see Lustig et al. (23).

Preliminary validity of the Klenico self-report module has been confirmed in a study comparing the results of Klenico to the gold-standard Structured Clinical Interview for DSM-IV (SCID) in an outpatient sample. Klenico led to similar diagnostic results as the SCID, demonstrating criterion validity. Moreover initial convergent and divergent validity were shown (Reutimann et al., in press^1^). Evidence for factorial validity and sensitivity to change for the Klenico depression domain was demonstrated in a sample of inpatients from the same clinic as the sample used in the current study (Reutimann et al., submitted manuscript^2^).

The current study seeks to confirm the above-mentioned preliminary results in a larger sample and with more fine-grained analyses. The aim of this study was to test construct validity in a routine clinical inpatient sample by focusing on both self-report and clinical validation modules of Klenico. In the present study, all those Klenico domains were tested for which congruent routine self-report questionnaires were available.

## 2. Methods

### 2.1. Procedure

Data were collected between 2019 and 2022 in a psychotherapeutic inpatient clinic that treats patients with a wide range of mental disorders, particularly those with eating disorders, OCD, and depressive disorders. Patients receive cognitive-behavioral therapy both individually and in group settings.

All patients underwent the clinic's routine diagnostic procedure, from which the data in this study originated. The Klenico system was temporarily integrated into this routine as an add-on to usual care.

Upon admission, patients were informed about the procedures by the clinical staff and asked for written consent that their data may be used for scientific studies. On the day of admission, patients received their routine diagnosis according to ICD-10 after an initial unstructured interview by the clinic's mental health professionals. Afterwards, patients were given routine self-report questionnaires (BDI-II, PHQ, and BSI were given to all patients; Y-BOCS, OCI-R, and EDE-Q disorder specific) and access to the Klenico self-report module by clinic staff.

Within the first two weeks of their stay, patients completed the routine questionnaires and responded to the Klenico self-report module on a device of their choice (computer or tablet). Finally, the clinic's mental health professionals completed the Klenico clinical validation module with each patient. The timing of the clinical validation depended on the availability of the professionals.

Before conducting their first Klenico clinical validation, professionals received an introduction and a demo on how to use the system, as well as an explanation of how to interpret and explain the results. Most of the professionals were psychotherapists in training for cognitive behavioural therapy under supervision with a minimum of one year professional experience. In addition, a co-therapist with several years of professional experience was part of the team. All of these professionals exhibited the required competence to make diagnoses in Germany. On average, the professionals conducted 2–3 clinical validations per week, and the average duration of the clinical validations was 66 min.

### 2.2. Measures

#### 2.2.1. Beck depression inventory (BDI-II)

The BDI-II (24) is a valid and reliable questionnaire (25) to assess depressive symptoms. It consists of 21 items on a four-point Likert scale ranging from 0 (symptom absent) to 3 (severe symptom) with a maximum score of 63. The sum score was calculated for each participant. Cronbach's alpha was 0.75.

#### 2.2.2. Patient health questionnaire (PHQ-D)

The PHQ-D (26) is based on the Diagnostic and Statistical Manual of Mental Disorders, 4th Edition (DSM-IV) and has good psychometric properties (27). Its short form consists of nine items for depression (PHQ-9), seven items for generalized anxiety (PHQ-GAD-7), both with a four-point Likert scale ranging from 0 (not at all) to 3 (nearly every day), and 15 items for somatoform symptoms (PHQ-15) with a three-point Likert scale ranging from 0 (not bothered at all) to 2 (bothered a lot). Sum scores of PHQ-9 (maximum score = 27), PHQ-15 (maximum score = 30), and PHQ-GAD-7 (maximum score = 21) were calculated for each participant. Cronbach's alpha was 0.84, 0.80 and 0.83, respectively.

#### 2.2.3. Brief symptom inventory (BSI)

The BSI (28) consists of 53 items on nine subscales (somatization, obsession-compulsion, interpersonal sensitivity, depression, anxiety, hostility/aggression, phobic anxiety, paranoid ideation, and psychoticism) with a five-point Likert scale ranging from 0 (not at all) to 4 (extremely). The BSI shows satisfactory psychometric properties. Its factor structure is questionable (29). The sum score was calculated for each subscale for each participant. Cronbach's alpha was between 0.70 and 0.83.

#### 2.2.4. Yale-Brown obsessive compulsive scale (Y-BOCS)

The Y-BOCS (30) consists of a symptom checklist to quantify different obsessions and actions. Impairment in everyday life, suffering, and resistance and control over the symptoms are recorded separately for obsessions and compulsions on a five-point scale ranging from 0 (no symptoms) to 4 (extreme symptoms). The sum score was calculated for each participant, with a maximum of 40. The Y-BOCS is a valid and reliable instrument for assessing OCD symptoms (31, 32). Cronbach's alpha was 0.76.

#### 2.2.5. Obsessive-compulsive inventory (OCI-R)

The OCI-R (33) is a self-assessment tool with 18 items recording the most common OCD symptom areas on six subscales (washing, checking, ordering, neutralizing, obsessing, and hoarding) with a five-point Likert scale ranging from 0 (not at all) to 4 (extremely). It is a reliable and valid questionnaire (34). The sum score was calculated (maximum score = 168). Cronbach's alpha was 0.90.

#### 2.2.6. Eating disorder examination-questionnaire (EDE-Q)

The EDE-Q (35), comprising 28 items and four subscales (restraint, eating concern, weight concern, shape worries), is a screening tool that measures the range and severity of eating disorder symptoms on a seven-point scale, ranging from 0 (never) to 6 (every day). It has good psychometric properties (36). However, the number of its underlying factors is questionable (37). The sum score was calculated for each participant, with a maximum of 168. Cronbach's alpha was 0.76.

#### 2.2.7. Routine ICD-10 diagnoses

After the initial, unstructured interview, the treating mental health professionals provided diagnoses based on the patients' current symptoms and coded them according to ICD-10. Compared to structured interviews, unstructured interviews lack validity (38), which can affect the quality of the diagnoses made (15).

#### 2.2.8. Klenico assessment

In Klenico's self-report module, patients were presented with 379 items based on ICD-10. The items were randomly displayed in groups of nine per screen, of which patients selected applicable ones (i.e., the symptoms that applied to them). Patients then rated the symptom severity by dragging and dropping the previously selected items onto a visual analogue scale ranging from 1 to 100. In the clinical validation module, mental health professionals confirmed or re-rated patients' self-reports. They also assessed additional items that required external perspective and therefore, did not occur in the self-report module (97 items). In this study, all those Klenico domains were tested for which congruent routine self-report questionnaires were available (anxiety disorders, depressive disorders, eating disorders, OCD, psychotic disorders, and somatoform disorders). These domains contain a total of 176 items in the self-report module. The mean score for each Klenico domain tested was calculated.

### 2.3. Sample

Anonymized data from 495 adults [*n *= 336 female, *n* = 159 male, mean age = 38.9 (*SD* = 15.0)] were used. Of these, 443 datasets included the full assessment because 52 patients only completed the self-report module.

Table 1 reports the frequencies of the given routine ICD-10 diagnoses by the clinic's mental health professionals using routine diagnostic procedures. Most frequent were depressive disorders followed by eating disorders. The mean number of diagnoses was 1.9.

**Table 1:** Frequencies of ICD-10 diagnostic categories.

Diagnostic Group	ICD-10 Code	Frequency	Percent
Depressive disorders	F32.x–34.x	390	42.3
Eating disorders	F50.xx	134	14.5
Somatoform disorders	F45.xx	91	9.9
Anxiety disorders	F40.xx–41.x	84	9.1
Reaction to severe stress, and adjustment disorders	F43.x	69	7.5
Obsessive-compulsive disorder	F42.x	56	6.1
Disorders of adult personality and behavior	F6x.xx	50	5.4
Behavioral and emotional disorders with onset usually occurring in childhood and adolescence	F9x.xx	15	1.6
Mental and behavioral disorders due to psychoactive substance use	F1x.x	12	1.3
Dissociative (conversion) disorders	F44.xx	8	0.9
Nonorganic sleep disorder	F51.x	5	0.5
Bipolar affective disorder	F31.x	4	0.4
Disorders of psychological development	F8x.xx	3	0.3
Other mental disorders due to brain damage and dysfunction and to physical disease	F06.x	1	0.1
Schizoaffective disorders	F25.x	1	0.1
Other neurotic disorders	F48.x	1	0.1

Groups were formed by assigning each individual routine ICD-10 diagnosis of each patient to the corresponding disorder domain. Multiple diagnoses are possible. Listed frequencies are relative to the number of total given diagnoses.

### 2.4. Statistical analysis

The software RStudio was used for data analysis (Version 2021.9.1.372, Boston, MA, USA). *P-*values < 0.05 were considered statistically significant.

Cronbach's alpha values of the tested Klenico domains were calculated for evaluation of internal consistency.

To determine convergent and divergent validity, a multitrait-multimethod (MTMM) matrix approach was used. This approach allows for concurrent inclusion of multimodal instruments and is a common method for evaluating psychological measurements (39).

To ensure a comprehensive evaluation of the Klenico system and obtain significant insights, all instruments used in the clinic's routine diagnostic procedures, such as the routine self-report questionnaires and routine ICD-10 diagnoses, were included in the analysis despite their limitations.

The MTMM contains three different types of correlations: Monotrait-heteromethod (associations between Klenico domains and corresponding self-report questionnaires as well as ICD-10 diagnoses), heterotrait-heteromethod (associations between Klenico domains and noncorresponding self-report questionnaires as well as ICD-10 diagnoses), and heterotrait-monomethod (associations between Klenico domains themselves).

By convention (40), convergent validity is demonstrated if the monotrait-heteromethod correlations are significantly high. Divergent validity is demonstrated if the heterotrait-monomethod correlations are lower than the monotrait-heteromethod correlations, the heterotrait-heteromethod correlations are lower than the monotrait-heteromethod correlations, and the correlation coefficients are comparably large, both within a method and between the different methods (40).

Pearson's correlation coefficients were calculated for associations between interval scaled variables, point-biserial correlations (r_pb_) were calculated for associations between interval scaled and dichotom variables, and Phi coefficients were calculated for associations between dichotom variables.

Correlation coefficients between 0.10 and 0.29 were considered as small, between 0.30 and 0.49 as medium, and ≥0.50 as large correlations (41).

## 3. Results

### 3.1. Internal consistency

Klenico domains demonstrated sufficient Cronbach's alpha coefficients of 0.70 or higher, except for the psychotic disorders (0.47) domain. For further details see Table 2.

**Table 2:** Internal consistencies of the tested Klenico domains.

Klenico Domains	Cronbach's alpha
Anxiety disorders	0.94
Depressive disorders	0.90
Eating disorders	0.85
OCD	0.81
Psychotic disorders	0.47
Somatoform disorders	0.79

OCD, obsessive-compulsive disorder.

### 3.2. Convergent and divergent validity of the Klenico domains

Correlations in the MTMM matrix must meet the following criteria to fulfill construct validity: **(I)** the monotrait-heteromethod correlations are significantly high; **(II)** the heterotrait-monomethod correlations are lower than the monotrait-heteromethod correlations; **(III)** the heterotrait-heteromethod correlations are lower than the monotrait-heteromethod correlations; and **(IV)** the correlation coefficients are approximately equal both within a method and between the different methods. Findings pertaining to these criteria are indicated in the descriptions below.

**Depressive disorders. (I)** This domain revealed significantly high monotrait-heteromethod correlations with the compared routine self-report questionnaire scales (ranging from r = 0.68 to r = 0.71) but a low correlation with the corresponding ICD-10 diagnosis of depression (r = 0.18). **(II), (III)** The monotrait-heteromethod correlations (ranging from r = 0.68 to r = 0.71) were all higher than both the heterotrait-monomethod correlations (ranging from r = 0.23 to r = 0.60) and the heterotrait-heteromethod correlations (ranging from r = **−**0.02 to r = 0.60), except for the correlation with the corresponding ICD-10 diagnosis.

**Eating disorders. (I)** This domain showed significantly high monotrait-heteromethod associations with the congruent ICD-10 diagnosis (r = 0.70) but no significant correlation with the EDE-Q (r = 0.26). **(II)**, **(III)** Apart from this nonsignificant association with the EDE-Q, all heterotrait correlations of the Klenico eating disorders domain (ranging from r = 0.00 to r = 0.32) are lower than the monotrait correlation with the compared ICD-10 diagnosis (r = 0.70).

**Anxiety disorders. (I)** This domain showed high monotrait-heteromethod correlations with the BSI (phobic) anxiety scales (ranging from r = 0.57 to r = 0.64), medium correlations with the BSI interpersonal sensitivity (r = 0.43) and the PHQ-GAD-7 (r = 0.45) scales, and weak correlations with the ICD-10 diagnosis (r = 0.23). **(II)** Its heterotrait-monomethod correlation with the Klenico somatoform disorders domain (r = 0.66) was higher than the monotrait-heteromethod correlations. **(III)** However, it showed particularly higher heterotrait-heteromethod correlations with the OCI-R (r = 0.61) than the monotrait-heteromethod correlations with the PHQ-GAD-7, the BSI (social) anxiety scales, and the ICD-10 diagnosis.

**Somatoform disorders. (I)** The somatoform disorder domain revealed significantly high monotrait-heteromethod correlations with the compared routine self-report questionnaire scales (ranging from r = 0.59 to r = 0.61) but low associations with the corresponding ICD-10 diagnoses (r = 0.21). **(II)** Again, the heterotrait-monomethod correlation between the somatoform disorders and anxiety disorders domains was higher (r = 0.66) than the monotrait-heteromethod correlations for the somatoform disorders domain. **(III)** However, it showed higher monotrait-heteromethod correlations (ranging from r = 0.59 to 0.61) than heterotrait-heteromethod correlations (ranging from r = 0.01 to r = 0.51), except for the association with the corresponding ICD-10 diagnosis (r = 0.21).

**OCD.** (**I)** This domain showed high correlations with the congruent ICD-10 diagnosis (r = 0.52) and the Y-BOCS (r = 0.73), a weak correlation with the BSI OCD scale (r = 0.29), and a nonsignificant correlation with the OCI-R (r = 0.31). **(II)** Further, the Klenico OCD domain revealed higher monotrait-heteromethod correlations (ranging from r = 0.52 to r = 0.73) than heterotrait-monomethod correlations (ranging from r = 0.23 to r = 0.39), except for the correlations with the OCI-R (r = 0.31) and BSI OCD (r = 0.29), which were lower. **(III)** Monotrait-heteromethod correlations (ranging from r = 0.52 to r = 0.73) were higher than heterotrait-heteromethod correlations (ranging from r = 0.02 to r = 0.34), except for the correlation with the BSI OCD scale (r = 0.29) and the nonsignificant correlation with the OCI-R (r = 0.31).

**Psychotic disorders. (I)** The Klenico psychotic disorders scale revealed only low monotrait-heteromethod correlations with the compared BSI psychoticism and paranoid ideation scales (ranging from r = 0.10 to r = 0.13) and no significant correlations with the given routine diagnosis (r = 0.03). **(II)**, **(III)** Its monotrait-heteromethod correlations with the congruent questionnaires and ICD-10 diagnoses were lower (ranging from r = 0.10 to r = 0.13) than the heteromethod correlations (ranging from r = 0.02 to r = 0.16).

**Correlations within and between methods. (IV)** The heterotrait-heteromethod correlations of the Klenico domains are comparable to the monotrait-heteromethod correlations for the respective domains. For example, the Klenico OCD domain showed a correlation coefficient of r = 0.23 with the Klenico depressive disorders domain and coefficients of r = 0.14, r = 0.14, and r = 0.18 with the BDI depression scale, the PHQ-9, and the BDI-II, respectively.

Correlation coefficients for all Klenico domains are presented in Table 3. The full MTMM can be found in the Supplementary Materials.

**Table 3:** Correlation coefficients of the tested Klenico domains with the compared methods of the MTMM approach. The full MTMM matrix can be found in the Supplementary Material*.*

		Klenico Domains					
		Depressive disorders	Anxiety disorders	Somatoform disorders	OCD	Eating disorders	Psychotic disorders
Klenico Domains	Depressive disorders	1.00					
	Anxiety disorders	0.60***	1.00				
	Somatoform disorders	0.46***	0.66***	1.00			
	OCD	0.23***	0.34***	0.23***	1.00		
	Eating disorders	0.29***	0.16***	0.16***	0.07	1.00	
	Psychotic disorders	0.26***	0.22***	0.19***	0.39***	0.07	1.00
Routine ICD-10 Diagnoses	Depressive disorders	0.18***	0.00	0.01	−0.08	−0.19***	0.06
	Anxiety disorders	−0.03	0.23***	0.11*	−0.02	−0.16***	−0.02
	Somatoform disorders	0.06	0.05	0.21***	−0.02	−0.11*	0.08
	OCD	−0.04	0.04	−0.05	0.52***	−0.10*	−0.03
	Eating disorders	0.06	−0.04	−0.08	0.02	0.70***	−0.03
	Schizoaffective disorder	−0.02	−0.05	−0.04	−0.02	0.00	0.03
BSI	Depression	0.68***	0.40***	0.23***	0.14**	0.27***	0.12*
	Anxiety	0.42***	0.57***	0.38***	0.27***	0.07	0.12*
	Phobic anxiety	0.34***	0.64***	0.34***	0.34***	0.09	0.15**
	Interpersonal sensitivity	0.52***	0.43***	0.24***	0.14**	0.32***	0.04
	Somatization	0.31***	0.48***	0.59***	0.23***	0.16***	0.06
	OCD	0.60***	0.40***	0.3***	0.29***	0.11*	0.15**
	Psychoticism	0.56***	0.41***	0.25***	0.27***	0.28***	0.13**
	Paranoid ideation	0.40***	0.28***	0.21***	0.09	0.14**	0.10*
PHQ	PHQ-9 (Depression)	0.70***	0.41***	0.29***	0.14**	0.29***	0.14**
	GAD-7 (Generalized anxiety)	0.52***	0.45***	0.30***	0.26***	0.10*	0.11*
	PHQ-15 (Somatization)	0.37***	0.44***	0.61***	0.12*	0.20***	0.08
BDI-II	Depression	0.71***	0.49***	0.31***	0.18***	0.30***	0.16***
OCI-R	OCD	0.39	0.61*	0.51*	0.31	−0.13	0.14
Y-BOCS	OCD	0.31	0.16	0.02	0.73**	0.00	−0.02
EDE-Q	Eating disorders	0.41**	0.43**	0.30*	0.17	0.26	0.22

BDI-II, beck depression Inventory II; BSI, brief symptom inventory; EDE-Q, eating disorder examination-questionnaire; ICD-10, international classification of diseases, 10th revision; OCD, obsessive-compulsive disorder; OCI-R, obsessive-compulsive inventory-revised; PHQ, patient health questionnaire; Y-BOCS, Yale-Brown obsessive compulsive scale.

## 4. Discussion

This current study aimed to confirm the construct validity of the Klenico assessment by using a MTMM approach to compare the results of the Klenico domain scores with standard self-report questionnaires and routine ICD-10 diagnoses in a large clinical inpatient sample.

### 4.1. Anxiety disorders

While the Klenico anxiety disorders domain demonstrated strong congruent associations with the BSI (phobic) anxiety scales, correlations with the BSI interpersonal sensitivity scale and the PHQ-GAD-7 were in the medium range, and the correlation with the congruent ICD-10 diagnosis was in the low range. The heterotrait-monomethod correlation between this domain and the Klenico somatoform disorders domain was higher than the correlations with the congruent questionnaire scales, and the correlations with the rest of the Klenico domains were lower. The heterotrait-heteromethod correlation with the OCI-R was in the high range and higher than the monotrait-heteromethod correlations, except for the correlation with the BSI phobic anxiety scale. Furthermore, the heterotrait-heteromethod correlations with the BDI-II and the BSI somatization scale were higher than the medium-range monotrait-heteromethod correlations with the BSI interpersonal sensitivity scale and the PHQ-GAD-7.

Reasons for the low associations of the anxiety disorders domain with the corresponding ICD-10 diagnosis might be inherent in routine diagnostic procedures. Diagnoses were made by the inpatient clinic professionals using unstructured interviews, which often lack validity and reliability, thus compromising the quality of the compared ICD-10 diagnoses (42). This counts for the anxiety disorders domain and all other Klenico domains tested. Therefore, the ICD-10 diagnoses are not used as a decisive criterion for the construct validity of the Klenico domains, but rather provide additional indications.

The same applies to the comparison to the BSI. The construction of the BSI was aimed at the assessment of symptom dimensions. Consequently, it demonstrates considerable heterogeneity in its underlying constructs and has questionable factorial validity (43). Therefore, the BSI is not used as a main criterion but delivers additional indications on construct validity. In the case of anxiety disorders, the BSI interpersonal sensitivity scale includes symptoms that reflect aspects of depression rather than of social anxiety disorder, which explains the medium-range correlation with the Klenico anxiety disorders domain. This is reflected by a recent study (29).

The medium-range correlation between the Klenico anxiety disorders domain and the PHQ-GAD-7 can be attributed to the fact that the PHQ was developed based on the diagnostic criteria of the DSM (26). However, the items included in the Klenico anxiety disorders domain are derived from the ICD-10 diagnostic criteria, which differ substantially from those in the DSM (44).

One possible explanation for the high correlation of the Klenico anxiety disorders domain with the OCI-R is that the OCI-R may measure certain aspects of anxiety. A previous validation study has already shown an association of the OCI-R total score with anxiety questionnaires, albeit in the medium range (45). Additionally, the OCD subscale of the OCI-R revealed large associations with a compared anxiety measure in another study (46).

Finally, the high heterotrait-monomethod correlation between the Klenico anxiety disorders and somatoform disorders domains and also the medium-range heterotrait-heteromethod correlation with the BSI somatization scale is not surprising, as both disorder domains are highly comorbid (47) and show substantial symptom overlap; anxiety disorders in particular are represented by a wide range of somatic symptoms (48). The same applies to the medium-range heterotrait-heteromethod correlation with the BDI-II because of the highly frequent comorbidity of anxiety and depressive disorders (49).

Overall, although results partially indicate construct validity for the Klenico anxiety disorders domain, this could not be definitively confirmed. The fact that the anxiety disorders were analyzed together as a whole domain and not as individual disorders may have influenced the results. Nonetheless, this summary analysis makes sense due to the partly shared symptomatology and etiology as well as the frequent comorbidities between the different anxiety disorders (50). However, the various disorders still have unique features and distinctions, which can limit a summary analysis (51, 52). Therefore, the anxiety disorders should be analyzed again individually in order to fully confirm the construct validity of the Klenico anxiety disorders domain.

### 4.2. Depressive disorders

The Klenico depressive disorders domain revealed high correlations with the compared depression measures (BDI-II and PHQ-9) but low correlations with the congruent ICD-10 diagnosis. Consequently, the monotrait-heteromethod correlations for this domain were higher than all the heterotrait correlations, with the exception of the nonsignificant monotrait-heteromethod correlation with the ICD-10 diagnosis.

Due to the high correlations with the well validated PHQ-9 and BDI-II and the low correlations with the noncongruent questionnaires, convergent and divergent validity are established. Accordingly, construct validity of the Klenico depressive disorders domain was demonstrated.

### 4.3. Eating disorders

The Klenico eating disorders domain revealed strong correlations with the ICD-10 diagnosis for eating disorders but low correlations with the congruent questionnaire, the EDE-Q. Consequently, while all heteromethod correlations were lower than the monotrait-heteromethod correlation with the ICD-10 diagnosis for eating disorders, they were higher than the monotrait-heteromethod correlation with the EDE-Q.

This nonsignificant correlation between the Klenico eating disorders domain and the EDE-Q is not striking. First, the EDE-Q does not include certain important ICD-10 diagnostic criteria, such as self-initiated vomiting, binge eating, amenorrhea, or loss of libido (53). And secondly, previous studies have failed to establish a one-factor model of the EDE-Q (54), which challenges the calculation of an overall sum score.

Thus, the nonsignificant correlation of the EDE-Q sum score with the Klenico eating disorders domain does not compromise the domain's convergent or divergent validity. Based on the low correlations with the noncongruent questionnaires, divergent validity can be confirmed for the Klenico eating disorders domain. Even the domain's convergent validity is indicated by the high correlation with the ICD-10 eating disorders diagnosis, additional research is needed to compare the domain with other more congruent measurements than the EDE-Q to fully confirm its convergent validity.

### 4.4. Psychotic disorders

The Klenico psychotic disorders domain revealed low correlations with the two corresponding BSI scales, psychoticism and paranoid ideation, and a nonsignificant monotrait-heteromethod correlation with the ICD-10 diagnosis. Although the heterotrait-monomethod correlations with the other Klenico domains were in the medium range, they were higher than the monotrait-heteromethod correlations, except for the eating disorders domain. The heterotrait-heteromethod correlations were all nonsignificant or in the low range. However, the correlations with the BSI phobic anxiety and OCD scales, as well as with the PHQ-9 and BDI-II depression questionnaires, were minimally higher than the monotrait-heteromethod correlations, but all in the low range.

Again, the low correlations between the Klenico psychotic disorders domain and both BSI scales, paranoid ideation and psychoticism, can be attributed to their underlying constructs. These two scales, in particular, have been criticized for representing constructs too heterogeneously (55). Also, the low correlation with the ICD-10 diagnosis is not striking as there was only one corresponding diagnosis for the Klenico psychotic disorders domain, namely schizoaffective disorder.

Although divergent validity for the Klenico psychotic disorders domain is not met based on the MTMM, it was suggested by the finding that all correlations with the noncongruent questionnaires were low. Nevertheless, the convergent validity of the Klenico psychotic disorders domain still needs to be demonstrated in a more suitable sample, for instance from a psychiatric setting, and against more standard questionnaires representing more appropriate constructs.

### 4.5. OCD

The Klenico OCD domain revealed high monotrait-heteromethod correlations with the corresponding ICD-10 diagnosis and the Y-BOCS. However, although significant, the associations between the Klenico OCD domain and the BSI OCD scale were low, and correlations with the OCI-R were nonsignificant. All monotrait-heteromethod and heterotrait-monomethod correlations were lower than the monotrait-heteromethod correlations with the ICD-10 diagnosis and the Y-BOCS, but higher than the monotrait-heteromethod correlations with the BSI OCD scale and the OCI-R.

The low correlation of the Klenico OCD domain with the OCI-R can be attributed to this questionnaire's measured OCD construct. Earlier studies found only a medium correlation between the Y-BOCS and the OCI-R (45). This may be because the OCI-R, in contrast to the Klenico OCD domain, tends to measure the stress related to specific OCD conditions rather than measuring overall symptom severity based on diagnostic criteria (56).

The apparent lack of associations between the Klenico OCD domain and the BSI OCD scale can be explained by the properties of the BSI. Half of the items of the OCD scale measure nonspecific symptoms that can also be attributed to depression (57).

Taken together, neither the low correlation with the OCI-R nor the low correlation with the BSI OCS compromises the Klenico OCD domain's construct validity, as the results could be well reasoned on the properties and measured constructs of the two tools. The high correlations with the Y-BOCS, as well as the low correlations with the noncongruent questionnaires and diagnoses, therefore demonstrates its construct validity.

### 4.6. Somatoform disorders

The Klenico somatoform disorders domain showed high correlations with the congruent questionnaire scales (BSI somatization and PHQ-15) but low correlations with the corresponding ICD-10 diagnosis. Consequently, the monotrait-heteromethod correlations with the BSI somatization and the PHQ-15 were higher than all of the heterotrait-heteromethod correlations. Finally, the heterotrait-monomethod correlations were lower than the monotrait-heteromethod correlations, except for the high monotrait-heteromethod correlation with the Klenico anxiety disorders domain.

Again, the high heterotrait-monomethod correlation between the Klenico somatoform disorders and anxiety disorders domains can be explained by the frequent comorbidities between the two domains (47) and the wide range of somatic symptoms common in anxiety disorders (48).

Consequently, convergent and divergent validity are demonstrated according to conventions; therefore, construct validity of the somatoform disorders domain could be confirmed.

### 4.7. Internal consistency

All but one of the Klenico domains reached good internal consistency, providing evidence for reliability. The exception was the psychotic disorders domain. This might be explained by the fact that psychotic disorders were insufficiently represented in the sample, as patients with psychotic disorders will more likely be referred to psychiatric instead of psychotherapeutic clinics. It therefore requires further investigation.

### 4.8. Unstandardized diagnoses

As previously mentioned, low correlations between the Klenico anxiety disorders, depressive disorders, and somatoform disorder domains and their corresponding routine ICD-10 diagnoses should not undermine the construct validity of these domains. The low correlations are likely attributable to the quality of the diagnoses, which were based on unstructured interviews conducted by clinic staff. Previous research has indicated that such assessments may lack reliability and validity (38, 42), potentially resulting in incorrect diagnoses (15).

Although it is possible that the properties of the Klenico domains contribute to the low correlations, there are arguments against this. Firstly, the routine ICD-10 diagnoses also showed low or non-significant associations with other well-validated self-report measures, such as the BDI-II and PHQ subscales in our current analysis. Additionally, in a previous study, the Klenico domains showed criterion validity according to the SCID, the gold standard in diagnostics, indicating Klenico's high diagnostic potential (24).

The high correlations observed between the Klenico eating disorders and OCD domains and their respective ICD-10 diagnoses suggest that the quality of diagnoses made via unstructured interview may vary between different disorders and depend on the experience and specialization of the clinical professional.

Consequently, the results of this study once again implies the weaknesses of diagnoses using unstructured interviews and emphasizes the need for standardized assessments. However, since structured tools such as the SCID are rarely used in clinical practice—because, among other reasons, they are considered too time-consuming (20)—new solutions are needed. Diagnostic software such as Klenico, which enables efficient diagnostic procedures, can therefore offer a solution.

### 4.9. Competence and training of psychotherapists

The standard diagnosis and the Klenico clinical validation were conducted by psychotherapists (in training), which is a common practice in Germany. Psychotherapists undergo rigorous training and have a licence and therefore, have the competence to diagnose mental disorders.

In Germany, the education of psychotherapy is regulated by law, and the professional title is protected. The provision of psychotherapy is limited to certified psychologists and physicians, including psychiatrists, who have completed extensive specialized training and received official recognition in the field of psychotherapy. Psychologists, for instance, must successfully complete a comprehensive three-year full-time practical training program, totaling 4,200 h. This training includes a year-long internship at an accredited psychiatric institution, six months of clinical work at an outpatient facility, a minimum of 600 h of supervised psychotherapy in an outpatient setting, and attendance of at least 600 h of theoretical seminars.

These characteristics distinguishes Germany from many other countries in Europe and around the world. For instance, in the UK the title of psychotherapist is not protected, and the training is not regulated by law. Instead, professional associations set their own standards for training. As a result, a more diverse range of professional backgrounds, including nursing, social work, and teaching, can access psychotherapist training in the UK and similarly in other countries like Australia.

### 4.10. Implications

Overall, the results revealed evidence for construct validity of the Klenico depressive disorders, OCD, and somatoform disorders domains and for reliability of the majority of the tested Klenico domains. Exceptions were the eating disorders and psychotic disorders domains, where only divergent validity was indicated, and the anxiety disorders domain. Validity of these domains needs further confirmation.

Consequently, this study partially confirms the results of previous studies of construct validity of the Klenico in psychotherapeutic in- and outpatient settings (24, 25) and extends previous findings to demonstrate validity of the entire Klenico system, including both the self-report and clinical validation module. Accordingly, Klenico offers high potential for improving diagnostic accuracy by utilizing standardized procedures. Furthermore, the inclusion of all relevant mental disorders can support mental health professionals in comprehensive diagnostics and therefore help reliably identify comorbidities. This is crucial as comorbid mental disorders are highly frequent (58) but often missed in clinical practice, again due to the use of unstructured interviews (15). Thus, only comprehensive, valid diagnoses, which Klenico enables, can lead to effective and evidence-based therapeutic interventions and, ultimately, to successful therapeutic outcomes.

A major strength of the study is the large sample, collected in the routine diagnostic procedures of a psychotherapeutic inpatient clinic, thus reflecting the implementation of the Klenico system in everyday clinical practice while presenting high ecological validity. For those reasons, results are well generalizable to psychotherapeutic inpatient and comparable settings. However, since there are likely fewer patients with severe mental illness in psychotherapeutic compared to psychiatric settings, this may limit the generalizability to psychiatric settings, and therefore requires further investigation.

### 4.11. Limitations

Due to the design of the MTMM approach and the types of routine self-report questionnaires available for comparison, the individual disorders had to be grouped into domains for this study and not all the Klenico domains were tested. The analysis of the whole domains may have led to certain limitations. This applies in particular to anxiety disorders, which should be examined again separately.

Secondly, since the data for this study were collected in routine diagnostic assessments, we had no influence on which questionnaires were used in the procedure. Therefore, appropriate questionnaires were not available for all Klenico domains, which partially limited the demonstration of convergent validity. For instance, the BSI, which has heterogeneity in the underlying constructs and questionable validity for certain scales, was used for the analysis. Therefore, the conclusions drawn from its comparison must be taken with caution. The same applies to the routine ICD-10 diagnoses used in the comparison because, as already discussed, they may lack reliability and validity given their basis on unstructured interviews. Further investigation is needed for certain Klenico domains, such as addictive disorders or ADHD, due to the lack of suitable questionnaires available for assessment.

Finally, although testing Klenico under real-life clinical conditions had the great advantage of high ecological validity, it also meant that the data collection was not standardized. Therefore, it is not known if and how many patients dropped out of treatment and how the different settings used in the study may vary. Because the interrater agreement of the clinical validation module could not be calculated, heterogeneity in the diagnostic quality of the individual professionals is possible.

## 5. Conclusion

In summary, this analysis confirms the earlier preliminary findings for the Klenico self-report module and demonstrates construct validity for the entire Klenico system. These results demonstrate that Klenico has the potential to close the gap of unstandardized diagnostic procedures. The system can support mental health professionals in making accurate diagnoses, which, in turn, can help them to select appropriate, evidence-based treatment for their patients.

Regarding future research, Klenico domains—in particular the eating disorders and psychotic disorders domains—need to be tested against more appropriate questionnaires to confirm their convergent validity. Moreover, future studies should focus on structural models to generate further insights into the factorial and structural validity of the Klenico system.

## Data Availability

The raw data supporting the conclusions of this article will be made available by the authors, without undue reservation.

## Footnotes

^1^Reutimann S, Milanovic D, Gulewtisch MD, Augsburger M. Preliminary validation of the Klenico diagnostic software self-report module through comparison with the diagnostic gold standard in an outpatient routine clinical sample. *Health Psychol Behav Med*. (in press).

^2^Reutimann S, Steiner J, Hübscher N, Milanovic D, Voderholzer U, Meule A, et al. Assessing construct validity and sensitivity to change of the Klenico depression scale. (2022). Manuscript submitted for publication.
